# A139 ONE OF THESE THINGS IS NOT LIKE THE OTHER: A CURIOUS CASE OF DISCORDANT ENDOSCOPIC OPTICAL EVALUATION

**DOI:** 10.1093/jcag/gwac036.139

**Published:** 2023-03-07

**Authors:** N T Aboalfaraj, N Shahidi, M Tomaszewski, S Jiang, W Xiong

## Abstract

**Background:**

Optical evaluation has become the cornerstone of early gastrointestinal neoplasia management as it informs therapeutic decisions between minimally invasive endoscopic resection techniques and surgery.

**Purpose:**

We report an unusual case of a subepithelial gastric inlet patch (GIP) with optical evaluation mimicking early squamous neoplasia

**Method:**

Case Presentation:

A 74-year-old male was referred for epigastric discomfort. On esophagogastroduodenoscopy, a 10-mm GIP was identified at 23cm from the incisors. Furthermore, a 15-mm polypoid lesion was identified at 25cm from the incisors on the contralateral wall. On optical evaluation, it was believed to be atypical but suspicious for squamous neoplasia (Figure 1). Histopathology of the lesion revealed a stratified squamous epithelium with an underlying focus on glandular mucosa. After a multidisciplinary team review, endoscopic submucosal dissection was performed, given the atypical optical characteristics of the lesion.

**Result(s):**

Histopathology of the lesion identified subepithelial gastric cardia-type mucosa with focal extension into the muscularis mucosae, most consistent with a subepithelial GIP. No neoplasia was identified.

**Image:**

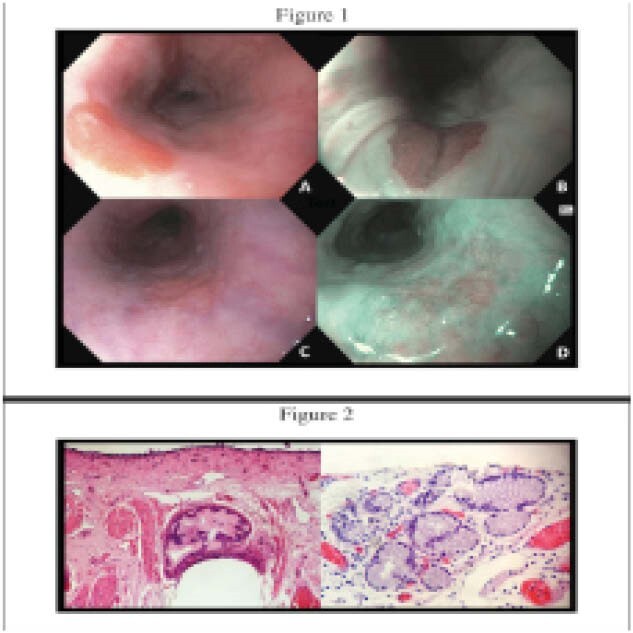

**Conclusion(s):**

We describe an unusual case of subepithelial GIP. With the widespread availability of high-definition endoscopes equipped with image-enhanced endoscopy, this case highlights the optical characteristics of this likely underappreciated endoscopic finding.

**Please acknowledge all funding agencies by checking the applicable boxes below:**

None

**Disclosure of Interest:**

None Declared

